# Host-Like Conditions Are Required for T6SS-Mediated Competition among Vibrio fischeri Light Organ Symbionts

**DOI:** 10.1128/mSphere.01288-20

**Published:** 2021-07-21

**Authors:** Lauren Speare, Madison Woo, Katherine M. Bultman, Mark J. Mandel, Michael S. Wollenberg, Alecia N. Septer

**Affiliations:** a Department of Earth, Marine, and Environmental Sciences, University of North Carolina at Chapel Hill, Chapel Hill, North Carolina, USA; b Department of Medical Microbiology and Immunology, University of Wisconsin, Madison, Wisconsin, USA; c Department of Biology, Kalamazoo College, Kalamazoo, Michigan, USA; University of Iowa

**Keywords:** *Aliivibrio fischeri*, type VI secretion, aggregation, *Euprymna scolopes*, pH, viscosity

## Abstract

Bacteria employ diverse competitive strategies to enhance fitness and promote their own propagation. However, little is known about how symbiotic bacteria modulate competitive mechanisms as they compete for a host niche. The bacterium Vibrio fischeri forms a symbiotic relationship with marine animals and encodes a type VI secretion system (T6SS), which is a contact-dependent killing mechanism used to eliminate competitors during colonization of the Euprymna scolopes squid light organ. Like other horizontally acquired symbionts, V. fischeri experiences changes in its physical and chemical environment during symbiosis establishment. Therefore, we probed both environmental and host-like conditions to identify ecologically relevant cues that control T6SS-dependent competition during habitat transition. Although the T6SS did not confer a competitive advantage for V. fischeri strain ES401 under planktonic conditions, a combination of both host-like pH and viscosity was necessary for T6SS competition. For ES401, high viscosity activates T6SS expression and neutral/acidic pH promotes cell-cell contact for killing, and this pH-dependent phenotype was conserved in the majority of T6SS-encoding strains examined. We also identified a subset of V. fischeri isolates that engaged in T6SS-mediated competition at high viscosity under both planktonic and host-like pH conditions. T6SS phylogeny revealed that strains with pH-dependent phenotypes cluster together to form a subclade within the pH-independent strains, suggesting that V. fischeri may have recently evolved to limit competition to the host niche.

**IMPORTANCE** Bacteria have evolved diverse strategies to compete for limited space and resources. Because these mechanisms can be costly to use, their expression and function are often restricted to specific environments where the benefits outweigh the costs. However, little is known about the specific cues that modulate competitive mechanisms as bacterial symbionts transition between free-living and host habitats. Here, we used the bioluminescent squid and fish symbiont Vibrio fischeri to probe for host and environmental conditions that control interbacterial competition via the type VI secretion system. Our findings identify a new host-specific cue that promotes competition among many but not all V. fischeri isolates, underscoring the utility of studying multiple strains to reveal how competitive mechanisms may be differentially regulated among closely related populations as they evolve to fill distinct niches.

## INTRODUCTION

Bacteria experience dramatic changes in their physical and chemical environment as they transition from free-living to host-associated states. For example, host tissues are often coated in a highly viscous mucus layer, have low levels of iron, and can vary significantly in pH and osmolarity, compared to environmental conditions (1, 2). These host-specific cues can prompt cells to alter their behavior and enhance their fitness for a host niche, which promotes successful colonization and persistence after initial infection (3–6).

Because space and resources are often limited within desirable host colonization sites, bacteria have also evolved diverse strategies to outcompete other potential colonizers and contend with resident communities. One such mechanism is the type VI secretion system (T6SS), a contact-dependent nanoweapon that delivers effector proteins directly into target cells through a molecular syringe-like apparatus (7–9). T6SSs have been found encoded in the genomes of pathogenic (10–14) and commensal microbes (15–20), and recent evidence suggests that they are active in host microbiomes (15, 20, 21). Moreover, because the T6SS syringe is a large multisubunit protein structure that is predicted to be energetically costly to assemble and fire (22–24), many bacteria use regulatory strategies to restrict T6SS activity to environments where its benefit is thought to outweigh its cost.

Previous studies suggest that host-specific cues may serve as important signals to activate T6SSs for competition in the host. For example, the plant pathogen Agrobacterium tumefaciens induces T6SS gene expression when transferred from soil to acidic medium that mimics plant wound sites (25). Iron-limiting conditions found in host tissue induce transcription of T6SS genes in Pseudomonas aeruginosa (26) and enteroaggregative Escherichia coli (27), while bile salts modulate T6SS function of Salmonella enterica (28). Vibrio parahaemolyticus T6SS encoded on chromosome II (T6SS2) is more active in the lower-salt conditions found in many marine animal hosts compared to seawater (11). Moreover, a number of studies have documented strain-specific regulation of T6SSs for different isolates of the same bacterial species (29, 30). For example, environmental Vibrio cholerae isolates have a constitutively active T6SS (31–33), compared to clinical isolates such as the pandemic V. cholerae strain C6706, which modulates T6SS gene expression in the presence of mucins and bile salts (34). Thus, examining the host-specific cues that regulate T6SS activity in different isolates of diverse species will provide valuable insight into how bacterial populations have evolved to compete for distinct niches. However, few studies have examined how environmental and host-specific cues regulate T6SS activity of beneficial bacterial symbionts.

The bioluminescent marine bacterium Vibrio fischeri, which forms a symbiotic relationship with the Hawaiian bobtail squid, Euprymna scolopes, is a tractable model to explore conditional regulation of the T6SS in the context of a mutualism. When *E. scolopes* squid hatch, the light organs of these aposymbiotic juveniles are colonized by planktonic V. fischeri within hours (35). Although many strains of V. fischeri are found in seawater, adult *E. scolopes* light organ populations are typically composed of only a few strains (36), suggesting that different symbiotic genotypes compete for the limited space within this host habitat. Indeed, recent work has revealed that V. fischeri uses several competitive mechanisms during host colonization (37–40), including a strain-specific T6SS on chromosome II (T6SS2) (15, 41). T6SS2 prevents incompatible strains from co-occupying the same crypt within the squid light organ (15), thus controlling the strain diversity within colonization sites and spatially separating symbiont genotypes in the host. Like other horizontally acquired bacteria, V. fischeri experiences substantial changes in the physical and chemical environment during host colonization. Therefore, we hypothesized that these environmental changes serve as host-specific cues to regulate T6SS2 activity.

To test this hypothesis, we probed three environmental conditions that differ between the water column and the *E. scolopes* light organ: pH, viscosity, and osmolarity. Although the juvenile *E. scolopes* light organ is covered in a highly viscous and mildly acidic (pH ∼6.5) mucus matrix and contains a viscous, neutral (pH ∼7.5) lumen within the crypt spaces (42–49), the water column around coastal O’ahu is a lower-viscosity environment with a more alkaline pH of ∼8.2. Moreover, most cephalopods have hyperosmotic tissues compared to seawater (50, 51), which has an osmolarity of ∼1,000 mosM (50). Using a high-viscosity liquid medium (hydrogel) model to mimic the viscosity of host mucus, we recently discovered that the transition from lower to higher viscosity activates T6SS2 in a model *E. scolopes* light organ isolate, V. fischeri strain ES401 (41). Here, we expanded upon this work to test additional cues that impact T6SS2-mediated competition. We identified a new host-specific cue that controls bacterial competition among diverse V. fischeri strains, and our findings underscore the importance of studying multiple strains to reveal how competitive mechanisms are differentially regulated among closely related populations as they evolve to fill distinct niches.

## RESULTS AND DISCUSSION

### ES401 outcompetes ES114 under host-like conditions.

To identify additional host-specific cues that regulate T6SS2-mediated competition, we examined competitive outcomes between V. fischeri strains that were coincubated in environmental and host-like pH, viscosity, and osmolarity conditions. We performed coincubation experiments, as described previously (41, 52), in lower-viscosity liquid Luria-Bertani salt (LBS)medium (liquid) and higher-viscosity medium (hydrogel) buffered at pH 8.2 (seawater), 7.5 (crypt lumen), or 6.5 (light organ mucus) with an osmolarity of either ∼790 mosM (slightly diluted seawater) or ∼1,300 mosM (host like). Hydrogel medium was made by supplementing liquid LBS medium with the water-soluble polymer polyvinylpyrrolidone (PVP-360) to create a hydrogel with a viscosity within the range of mucus (∼152 cP). PVP hydrogel has been used to study bacterial motility (53), and we previously showed that its use does not impact the growth of V. fischeri (41).

**FIG 1:**
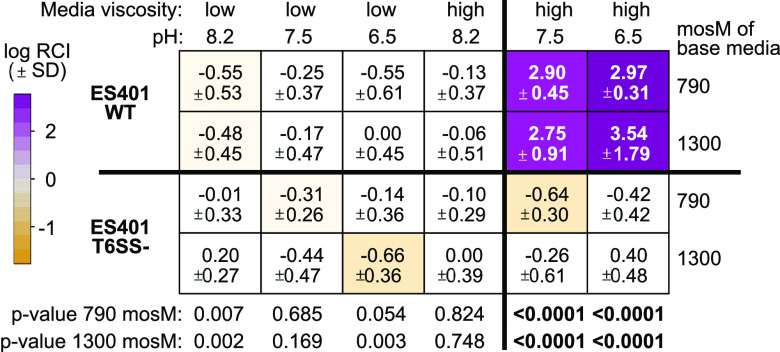
ES401 outcompetes ES114 in host-like pH and viscosity in a T6SS2-dependent manner. Heat map displaying results of coincubation assays between the ES114 (target) and ES401 (inhibitor) wild type (WT) or *tssF_2* mutant (T6SS-). Results are average log relative competitive (RCI) values calculated by an ES401/ES114 ratio at the end of the experiment (12 h) divided by the ratio for these strains at the beginning of the experiment (0 h). Positive log RCI values indicate that ES401 outcompeted ES114 (magenta), and a log RCI of 0 indicates that neither strain outcompeted the other strain (white). Coincubations were performed in liquid LBS medium (low viscosity) or liquid LBS medium that was supplemented with 5% (wt/vol) PVP (high viscosity) at pH 8.2, 7.5, or 6.5 with a base osmolarity of 790 or 1,300 mosM. Bold *P* values at the bottom of the heat map indicate that log RCI values were significantly higher in experiments with wild-type ES401 than with the T6SS2 mutant under a given condition (Student's *t* test) and log RCI values are greater than 0 in experiments with wild-type ES401. Each experiment was performed three times, and combined data are shown (*n* = 12). Standard deviations are listed below the log RCI values.

For our competition assays, we selected two V. fischeri strains isolated from *E. scolopes* light organs as competitors: ES114 as the target strain, which does not encode the T6SS2 genomic island (GI) (15), and ES401 as the inhibitor strain, which encodes T6SS2 (15) and kills ES114 in a T6SS2-dependent manner (41). We coincubated these differentially tagged strains in each combination of viscosity, pH, and osmolarity conditions (12 treatments) for 12 h and enumerated CFUs for both strains in each treatment at the beginning and end of the experiment. To determine whether ES401 outcompeted ES114 in a given treatment, we calculated the log relative competitive index (RCI) values for each treatment. A log RCI value of 0 indicates no competitive difference between strains (Fig. 1, white), while a log RCI value greater than 0 indicates that ES401 outcompeted ES114 (magenta), and a log RCI value less than 0 indicates that ES114 outcompeted ES401 (yellow). The role of T6SS2 was determined by comparing the log RCI values for coincubations with the wild-type inhibitor strain to coincubations performed with a T6SS2 mutant, which lacks a functional baseplate component (*tssF_2*/*vasA_2*). We sought to identify conditions in which log RCI values were above 0 for the wild type and significantly greater in experiments with the wild-type inhibitor than the T6SS2 mutant, which would suggest that T6SS-mediated killing occurred.

Using this quantitative approach, we determined that ES401 requires specific pH conditions for the T6SS2-dependent competition in hydrogel that we previously described (41). Specifically, four treatments resulted in log RCI values greater than 0 for the wild-type inhibitor strain: high viscosity at pH 6.5 and 7.5 for both osmolarity conditions (Fig. 1). For all four conditions, the log RCI values were statistically significantly higher in competitions using the wild type than those with the T6SS2 mutant, with an effect size of ∼1,000-fold (Fig. 1). In the remaining eight treatments, log RCI values were not significantly different from 0 for wild-type or T6SS2 mutant coincubations (Fig. 1), indicating that the ES401 wild-type and T6SS2 mutant competed equally with ES114. We did not observe any significant differences in competitive outcomes between ∼790 mosM and ∼1,300 mosM treatments, suggesting that under these conditions, osmolarity does not affect competitive outcomes between ES401 and ES114. Because of this finding, all remaining experiments were performed at 790 mosM. Moreover, there were no significant differences between ES401 and ES114 growth rates under any given condition (Table S1), indicating that growth rate did not impact competitive outcomes under the conditions tested here. Taken together, these data indicate that the T6SS-mediated competition observed under high-viscosity conditions is also pH dependent.

10.1128/mSphere.01288-20.2 TABLE S1 Strain growth rates under environmental and host-like conditions. Growth rates were calculated from three independent experiments with four biological replicates per experiment (*n* = 12). There were no significant differences in ES114 versus ES401 growth rates in any given condition (Student’s *t* test, column 8). Download Table S1, DOCX file, 0.1 MB. Copyright © 2021 Speare et al. 2021 Speare et al. https://creativecommons.org/licenses/by/4.0/ This content is distributed under the terms of the Creative Commons Attribution 4.0 International license.

Our data reveal that ES401 engages is T6SS2-mediated competition under pH and viscosity conditions that are consistent with juvenile *E. scolopes* squid but not under conditions experienced by free-living cells in the water column (low viscosity, pH 8.2). These observations are consistent with our previous finding that ES401 T6SS2 is functionally inactive in liquid LBS medium but is active in hydrogel at pH 7.5 (41). Although there are many possibilities for how pH could impact the chemistry of our hydrogel medium, including modification of potential PVP-solute interactions, we reasoned that it was possible to use our hydrogel model to identify the relevant physiological changes underlying the pH-dependent effect on competition. We considered two hypotheses to explain how host-like conditions control T6SS2 competition: (i) viscosity and/or pH impacts the cell’s ability to express or assemble T6SS2 syringe components, and/or (ii) viscosity and/or pH modulates the cell-cell contact between ES401 and ES114 that is required for T6SS-mediated killing.

### Medium pH does not affect a T6SS2 transcriptional reporter or the ability to build sheaths.

**FIG 2:**
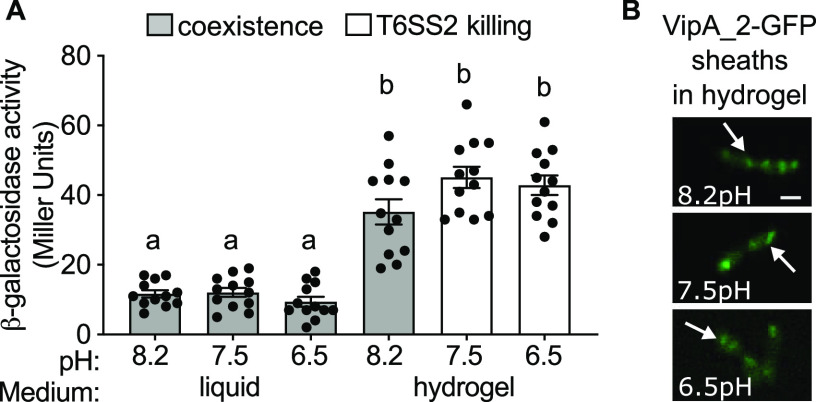
Medium pH does not affect T6SS2 transcriptional reporter or the ability to build sheaths. (A) β-Galactosidase assays were performed on ES401 cells harboring a T6SS2 reporter plasmid (pAG01) grown in LBS liquid medium (liquid) or LBS liquid medium supplemented with 5% (wt/vol) PVP (hydrogel) at pH 8.2, 7.5, or 6.5 for 12 h. White bars indicate conditions where T6SS2 killing was observed, and gray bars indicate conditions where no T6SS2 killing was observed (coexistence) in previous coincubation experiments. Letters indicate significantly different values (in Miller units) between medium conditions (one-way analysis of variance [ANOVA]; Sidak’s multiple-comparison test, *P < *0.0001). Experiments were performed three times, and combined data are shown (*n* = 12). Error bars indicate standard errors of the means (SEM). (B) Representative green fluorescent protein (GFP) images of ES401 harboring VipA_2-GFP incubated in hydrogel at the indicated pH for 2 h. Scale bar = 1 μm.

To test our first hypothesis that viscosity and pH impact T6SS2 expression, we utilized a *lacZ*-based promoter reporter for an essential T6SS structural gene, *hcp_2* (*tssD*), called P*_hcp_2_*. We previously determined that P*_hcp_2_* activity correlates with expression of the T6SS2 structural proteins, making it a good proxy for T6SS2 expression (41). To determine the extent to which viscosity and pH modulate T6SS2 expression, we performed β-galactosidase assays using strain ES401 containing the P*_hcp_2_*-*lacZ* reporter plasmid grown alone in liquid or hydrogel medium at pH 8.2, 7.5, or 6.5. Promoter activity was significantly higher in all hydrogel treatments than in the liquid treatments at each corresponding pH (Fig. 2A). We did not observe any significant difference in promoter activity between pH treatments in either liquid or hydrogel (Fig. 2A). These data indicate that T6SS2 promoter activity does not explain the decreased competitive ability of ES401 in pH 8.2 hydrogel.

We considered the possibility that medium pH may modulate posttranscriptional processes that inhibit ES401 T6SS2 assembly. To test this hypothesis, we used single-cell fluorescence microscopy to visualize cultures of ES401 carrying an inducible VipA_2-GFP expression vector, as described previously (41). VipA is one of two sheath subunits that are essential for constructing the T6SS syringe and by tagging VipA_2 with GFP, we can directly visualize T6SS assembly within live cells (15, 31, 41, 54). When ES401 cells expressing VipA_2-GFP were incubated in hydrogel, VipA_2-GFP sheaths were observed in each pH treatment and the majority of cells produced one or more sheaths (Fig. 2B), indicating that pH does not affect T6SS2 sheath assembly in hydrogel. Taken together, these data suggest that although high-viscosity conditions are essential for T6SS2 expression and sheath assembly, pH does not impact these functions. Therefore, we hypothesized that pH may affect the ability of ES401 to mediate the cell-cell contact necessary for T6SS-mediated killing in hydrogel.

### pH controls the cell-cell contact required for T6SS2 activity in hydrogel.

We previously showed that V. fischeri forms large, multistrain aggregates in hydrogel at pH 7.5 and that ES114 cells within these aggregates are in direct contact with ES401 and are therefore vulnerable to T6SS2-mediated killing (41). We reasoned that alkaline pH may alter the ability of inhibitor cells to aggregate, thereby reducing contact with the target strain, resulting in diminished competition in hydrogel at pH 8.2.

**FIG 3:**
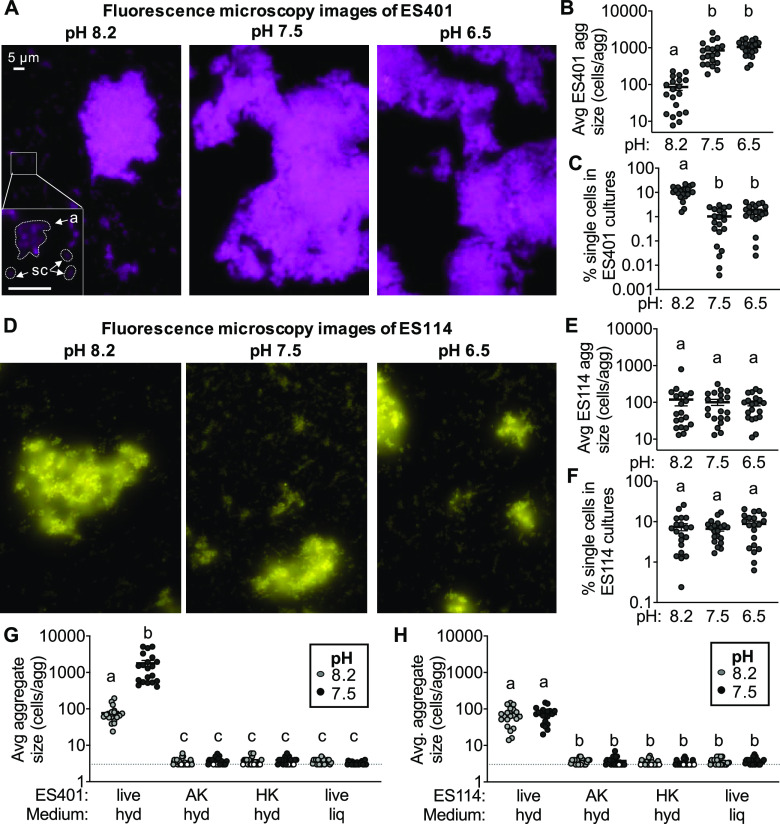
There is less biologically mediated cell-cell contact in hydrogel at pH 8.2 than pH 6.5 and 7.5 for strain ES401. Monocultures of ES401 or ES114 were incubated in pH 8.2, 7.5, or 6.5 hydrogel for 12 h. Representative single-cell fluorescence microscopy images of (A) ES401 and (D) ES114. (A, inset) The letter “a” indicates an example of an aggregate, and “sc” indicates examples of single cells. (B and E) Estimated average aggregate size in each pH treatment. Letters indicate significantly different estimated average aggregate size between pH conditions (one-way ANOVA; Sidak’s multiple-comparison test, *P < *0.0001). (C and F) Percentages of cells in each field of view that were not associated with aggregates (fewer than three cells touching) (% single cells). Single cells are defined as particles with an average area less than three V. fischeri cells (4.5 μm^2^). Letters indicate significantly different percentages of single cells between pH conditions (one-way ANOVA; Sidak’s multiple-comparison test, *P < *0.0001). (G and H) Estimated average aggregate size for monocultures of live, antibiotic-killed (AK), or heat-killed (HK) ES401 (G) or ES114 (H) cells that were incubated in hydrogel (hyd) or liquid (liq) medium with pH 8.2 (light gray) or 7.5 (dark gray) for 12 h. Antibiotic- and heat-killed cells were inoculated at a concentration 100-fold higher than live cells to account for no cell growth during the 12 h incubation. White data points indicate no aggregates were observed in the given field of view. Letters indicate significantly different estimated average aggregate size between pH conditions (one-way ANOVA; Sidak’s multiple-comparison test, *P < *0.0001). Each experiment was performed twice with two biological replicates and five fields of view (*n* = 20); each data point representants a single value for each biological replicate. Error bars indicate SEM.

To test the hypothesis that pH modulates cell-cell contact, we used fluorescence microscopy to visualize monocultures of fluorescently tagged ES401 and ES114 grown in pH 8.2, 7.5, or 6.5 hydrogel. Although ES401 aggregates were observed in each pH treatment (Fig. 3A), the estimated average aggregate size was significantly smaller for cultures grown in pH 8.2 than those at pH 7.5 and 6.5 (Fig. 3B). Moreover, the percentage of ES401 cells in each field of view that were not associated with aggregates, termed “single cells,” constituted a significantly higher percentage of the population in pH 8.2 hydrogel (Fig. 3C), suggesting that aggregate size is smaller because individual cells are not being recruited into aggregates as efficiently. In contrast, when we visualized monocultures of fluorescently tagged ES114 grown under the same conditions (Fig. 3D), we did not observe a difference in average aggregate size (Fig. 3E) or the percent single cells (Fig. 3F), suggesting that medium pH does not impact the aggregation ability of ES114. Furthermore, dead ES401 and ES114 cells (both heat- and antibiotic-killed cells) were unable to form substantial aggregates in hydrogel at pH 8.2 or 7.5 (Fig. 3G and H), resulting in a majority of the population as single cells (Fig. S1A and B). Together, these results suggest that (i) the strain-specific change in aggregation that we observed in response to changes in ambient pH is biologically mediated and not a result of physical crowding of particles by the PVP polymers, and (ii) there is less cell-cell contact in ES401 monocultures in pH 8.2 than in those at pH 7.5 and 6.5.

10.1128/mSphere.01288-20.4 FIG S1 Percent single cells for live and dead monocultures of ES401 and ES114. Estimated average aggregate size for monocultures of live, antibiotic-killed (AK), or heat-killed (HK) ES401 (A) or ES114 (B) cells that were incubated in hydrogel (hyd) or liquid (liq) medium with pH 8.2 (light gray) or 7.5 (dark gray) for 12 h. Antibiotic- and heat-killed cells were inoculated at a concentration 100-fold higher than live cells to account for no cell growth during the 12-hour incubation. White data points indicate that less than 0.1% of single cells were observed in the given field of view. Each experiment was performed twice with two biological replicates and five fields of view (*n* = 20); each data point representants a single value for each biological replicate. Error bars indicate SEM. Download FIG S1, EPS file, 0.2 MB. Copyright © 2021 Speare et al. 2021 Speare et al. https://creativecommons.org/licenses/by/4.0/ This content is distributed under the terms of the Creative Commons Attribution 4.0 International license.

**FIG 4:**
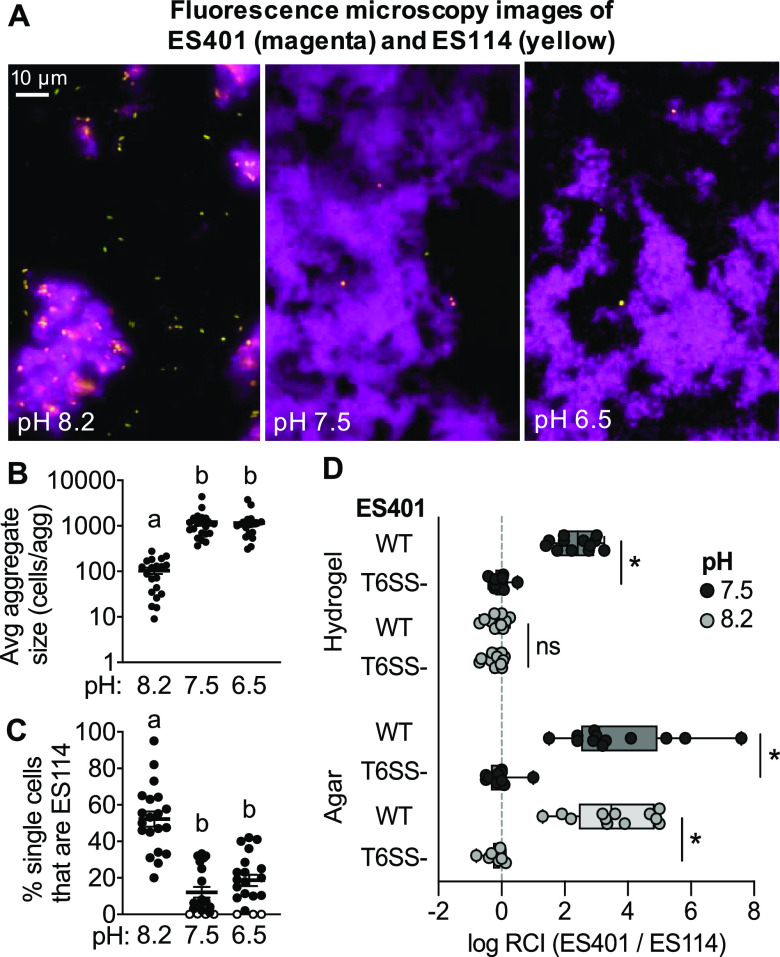
pH influences the cell-cell contact required for killing in hydrogel. Data in panels A to C were collected from cocultures of ES401 and ES114 that were incubated in pH 8.2, 7.5, or 6.5 hydrogel for 12 h. (A) Representative fluorescence microscopy images of ES401 (magenta) and ES114 (orange). (B) Estimated average aggregate size. Letters indicate significantly different estimated average aggregate size between pH conditions (one-way ANOVA; Sidak’s multiple-comparison test, *P < *0.0001). (C) Percent of single cells that are strain ES114. Letters indicate significantly different percentages of single cells that are ES114 between pH treatments (one-way ANOVA; Sidak’s multiple-comparison test, *P < *0.0001). Open circles indicate limit of detection (<1 single ES114 cell). Each experiment was performed twice with two biological replicates and five fields of view (*n* = 20); each data point representants a single value for each biological replicate. Error bars indicate SEM. (D) Results from coincubation assays between ES114 and the ES401 wild type (WT) or *vasA_2* mutant (T6SS-). Log relative competitive index (RCI) data were obtained from CFUs from 12-h coincubations performed on agar surfaces or in high-viscosity liquid medium (hydrogel) at either pH 8.2 (light gray) or pH 7.5 (dark gray). Log RCI values were calculated by an ES401/ES114 ratio at the end of the experiment (12 h) divided by the ratio of these strains at the beginning of the experiment (0 h), such that positive values indicate that ES401 outcompeted ES114. The vertical dashed line indicates a log RCI of 0 (strains competed equally). Asterisks indicate significantly higher log RCI values in experiments with the wild type than with the *vasA_2* mutant strain (Student’s *t* test, *P < *0.0001). Error bars indicate SEM. Experiments were performed at least three times, and combined data are shown (*n* = 12).

To quantify whether this reduced cell-cell contact between ES401 cells corresponds with reduced ES401-ES114 cell-cell contact, we visualized cocultures of differentially tagged ES401 and ES114 incubated in hydrogel at pH 8.2, 7.5, or 6.5 (Fig. 4A). Similar to the monocultures of ES401, average aggregate size was significantly smaller in pH 8.2 than under the other pH conditions (Fig. 4B). Moreover, when we quantified the proportion of strain type making up the single-cell fraction in each treatment, we found that more than 50% of single cells were ES114 in pH 8.2, while ES114 comprised less than 20% of the single-cell population at pH 7.5 and 6.5 (Fig. 4C). These data indicate that in hydrogel coculture, a larger proportion of ES114 cells are not in contact with ES401 cells in aggregates in pH 8.2 than at pH 7.5 and 6.5. Taken together, these observations suggest that medium pH can impact the extent to which inhibitor and target cells make contact in hydrogel and reveal that aggregate size is a good indicator of a strain’s ability to mediate the contact necessary for outcompeting a target strain in hydrogel.

To directly test whether the reduced cell-cell contact in hydrogel at pH 8.2 prevents ES401 from outcompeting ES114, we performed coincubation assays on agar surfaces at pH 8.2, where cells are forced into contact with one another. We predicted that if reduced cell-cell contact in pH 8.2 hydrogel prevents ES401 from outcompeting ES114, then ES401 will outcompete ES114 when cells are forced together on agar plates at pH 8.2. We collected CFUs from coincubations with ES114 and wild-type or T6SS2 mutant ES401 strains in hydrogel and on surfaces at pH 7.5 or 8.2 and calculated log RCI values. We chose to perform experiments at pH 7.5 only rather than both pH 6.5 and 7.5, because there were no significant differences in aggregation or killing ability for pH 6.5 and 7.5 in hydrogel medium. Consistent with our previous results, positive log RCI values were observed in hydrogel coincubations with wild-type ES401 at pH 7.5 but not pH 8.2 (Fig. 4D). In contrast, log RCI values were significantly greater for coincubations with the wild type relative to the T6SS2 mutant on agar surfaces at both pH 7.5 and 8.2 (Fig. 4D). These data indicate that pH conditions do not impact the ability of ES401 to outcompete ES114 in a T6SS2-dependent manner on agar surfaces where cell contact is forced and not biologically mediated. Together, these findings support a model whereby pH controls competitive outcomes in a high-viscosity, liquid environment by affecting cell-cell contact rather than modulating expression or assembly of sheath components.

This model is consistent with what is known about how V. fischeri responds to environmental pH during habitat transition, when cells experience an ∼50-fold increase in ambient hydrogen ions as they move from the water column (pH 8.2) to the host mucus (pH 6.5). Specifically, recent work indicates V. fischeri responds to this host-derived acidic cue by remodeling membrane physiology (55) and increasing levels of specific outer membrane proteins (49). Our data further suggest this host-like pH may alter cell surface components that promote cell-cell contact to facilitate T6SS-mediated competition.

### V. fischeri show a strain-specific ability to form large aggregates in hydrogel.

When quantifying aggregate sizes for ES401 and ES114, we noticed a stark difference in aggregation abilities for these two strains. Although ES401 formed large aggregates of ∼1,000 cells in hydrogel with pH 7.5 or 6.5 (Fig. 3A and B), strain ES114 formed only small aggregates of ∼100 cells under all pH conditions (Fig. 3D and E), similar to those observed for ES401 in pH 8.2 hydrogel. Although many strain-specific variations exist between ES401 and ES114, one major genotypic difference is the presence or absence of the T6SS2-encoding GI (15). In addition to encoding the structural genes for T6SS2, this GI also encodes many hypothetical proteins of unknown function (15), whose role in T6SS-mediated competition are not yet known.

**FIG 5:**
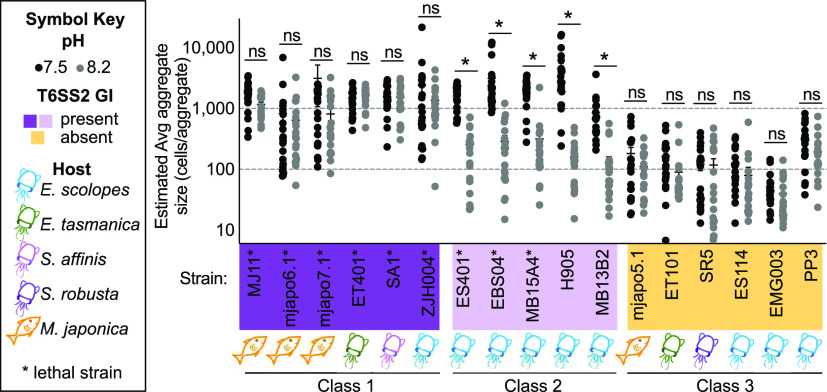
Estimated average aggregate size in hydrogel at pH 7.5 correlates with the presence of the T6SS2 GI. Estimated average aggregate size for monocultures of V. fischeri strains incubated in either pH 7.5 (dark gray) or 8.2 (light gray) hydrogel for 12 h. The presence of the T6SS2 GI is indicated by color: purple, present; orange, absent. Strains with a functional T6SS2 (lethal strains) are indicated with an asterisk after the strain name. Host source is indicated by symbol under each strain name, as shown in the key. *, *P < *0.0001 (Student's *t* test: ); ns, not significant (*P > *0.05). Strains are grouped into classes based on aggregation phenotype: class 1, ∼1,000 cells/aggregate in both pH treatments (dark purple); class 2, ∼1,000 cells/aggregate at pH 7.5 and ∼100 cells/aggregate at pH 8.2 (light purple); and class 3, ∼100 cells/aggregate in both pH treatments (orange). Each experiment was performed twice with two biological replicates and five fields of view, and combined data are shown (*n* = 20). Error bars indicate SEM.

Given that aggregate size is directly correlated with the ability of ES401 to use T6SS2 to outcompete a target strain in hydrogel, we asked how this strain-specific aggregation phenotype might impact the ability of other strains to use T6SS2 in hydrogel under different pH conditions. We therefore quantified the average aggregate size for an additional 15 V. fischeri strains with and without the T6SS2-encoding GI (15), including two newly identified inhibitor strains (Fig. S2), which can colonize the *E. scolopes* light organ or were isolated from the light organs of Euprymna tasmanica, and Sepiola affinis squid (15, 35, 36, 56–59), as well as the fish Monocentris japonica (60, 61). We observed three distinct classes of phenotypes: (i) class 1 strains, which formed large aggregates (∼1,000 cells/aggregate) under both pH conditions tested; (ii) class 2 strains, which formed large aggregates only at pH 7.5 and smaller aggregates (∼100 cells/aggregate) at pH 8.2; and (iii) class 3 strains, which formed only small aggregates under both pH conditions (Fig. 5). Class 1 and 2 strains, which are capable of forming large aggregates, all contain the T6SS2 GI, while class 3 strains (those that make only small aggregates) all lack the GI. Thus, the ability to make large aggregates correlates with the presence of the T6SS2-encoding GI, and pH-dependent effects on aggregation were observed only in a subset of these T6SS2 GI^+^ strains (Fig. 5). Based on these findings, and our previous results that show aggregate size correlates with killing ability in ES401 (Fig. 3 and 4), we hypothesized that if aggregate size is an indicator of a strain’s ability to mediate T6SS-dependent competition in hydrogel, then it can be used to predict which strains display a pH-dependent competitive ability.

10.1128/mSphere.01288-20.5 FIG S2 The *M. japonicus* light organ isolates mjapo6.1 and mjapo7.1 are lethal and encode T6SS2. Fluorescence microscopy images of GFP-tagged ES114 coincubated with eight V. fischeri strains isolated from *M. japonicus* light organs. Competitor strains are listed below each image. Bar = 2 mm. Coincubations with ES114 serve as a control for a nonlethal strain (ES114 is observed), and coincubations with MJ11 serve as a control for a lethal strain (ES114 is not observed) and are indicated with asterisks. Strains were screened for the presence/absence of the T6SS2 structural gene *tssF_2* and the housekeeping gene *recA.* FIG S2, EPS file, 0.1 MB Copyright © 2021 Speare et al. 2021 Speare et al. https://creativecommons.org/licenses/by/4.0/ This content is distributed under the terms of the Creative Commons Attribution 4.0 International license.

### Strain-specific killing response to pH correlates with aggregate size.

**FIG 6:**
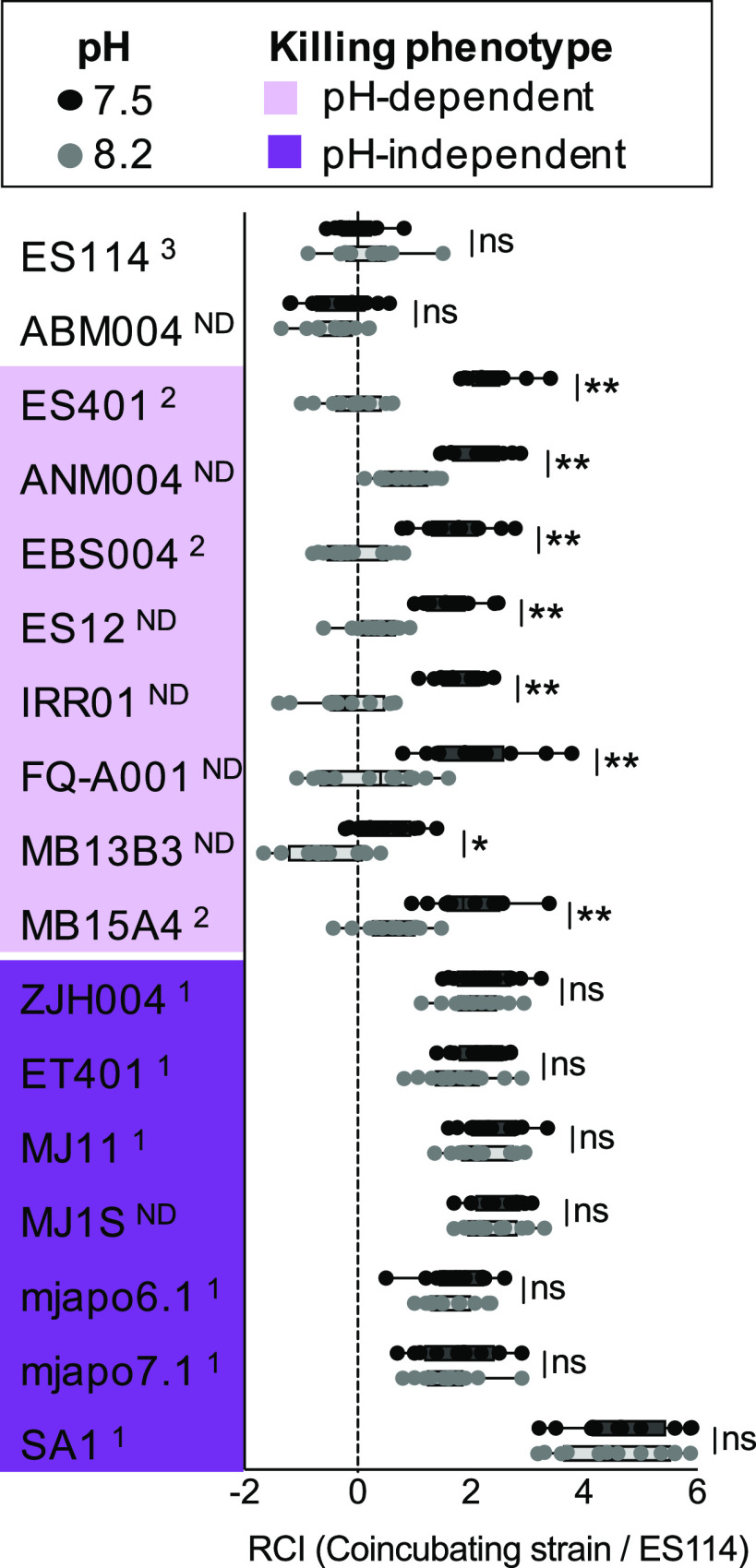
Aggregate size in hydrogel correlates with competitive phenotype in hydrogel. Results of coincubation assays between ES114 and lethal V. fischeri strains. Results are displayed as log RCI values calculated by a lethal-strain/ES114 ratio at the end of the experiment (12 h) divided by the ratio at the beginning of the experiment (0 h). Superscript numbers are strain classes, based on aggregation phenotype: class 1, ∼1,000 cells/aggregate in both pH treatments; class 2, ∼1,000 cells/aggregate in pH 7.5 and ∼100 cells/aggregate in pH 8.2; ND, aggregation phenotype not determined. Color indicates killing phenotype in hydrogel. *, *P < *0.0001 (Student's *t* test); ns, not significant. Each experiment was performed three times, and combined data are shown (*n* = 12). Error bars indicate SEM.

To directly test whether aggregate size is an indicator of killing ability in hydrogel, we performed coincubation assays in hydrogel at pH 7.5 or 8.2 using ES114 and all T6SS2+ class 1 and class 2 strains tested above, as well as six additional V. fischeri strains with a functional T6SS2. Control coincubations included ES114 with itself, and ES114 coincubated with another strain lacking the T6SS2 GI (ABM004) (15) or ES401. To evaluate whether a given strain outcompeted the target in a pH-dependent manner, we compared log RCI values between pH treatments for each strain. For half of the strains examined, including all tested class 2 strains, log RCI values were significantly greater at pH 7.5 that at pH 8.2 (Fig. 6) suggesting that, like ES401, these strains competed better against ES114 in pH 7.5 compared to pH 8.2. In contrast, the class 1 strains showed log RCI values that were not significantly different between pH treatments, but instead were significantly greater than zero in both pH treatments (Fig. 6), suggesting that these strains outcompete ES114 in hydrogel regardless of pH.

A subset of these strains were tested to determine whether competition was mediated by T6SS2. For class 2 strains that displayed a pH-dependent competitive phenotype, log RCI values were not significantly different between the wild type and the T6SS2 mutant in hydrogel at pH 8.2, suggesting that no T6SS2-mediated competition occurred (Fig. S3). However, for MJ11, a class 1 strain that outcompeted ES114 in both pH treatments, log RCI values were significantly higher in coincubations with the wild type relative to the T6SS2 mutant in hydrogel at pH 8.2 (Fig. S3), suggesting that T6SS2-mediated competition occurred. Thus, the ability of a strain to use T6SS2 to outcompete ES114 directly correlates with the strain’s ability to form large aggregates under a given condition (Fig. 5). Specifically, these data reveal that V. fischeri displays two distinct competitive phenotypes in response to medium pH: class 2 strains outcompete a target using T6SS2 in a pH-dependent manner, while class 1 strains outcompete a target using T6SS2 regardless of medium pH. Because this strain-specific competitive ability may have important implications for the propagation of class 1 and class 2 lineages in certain environments, we sought to further investigate the nature of this strain-specific behavior.

10.1128/mSphere.01288-20.6 FIG S3 Lethal V. fischeri strains outcompete ES114 in a T6SS2-dependent manner. A key for colors and symbols is at the top. Data point color indicates hydrogel pH: 7.5 (dark gray) or 8.2 (light gray). Data point shape indicates lethal strain genotype: circles, wild type; triangles, *tssF_*2-. Host source is indicated by color. Log RCI data for coincubations between ES114 and V. fischeri light organ isolates. Coincubations were performed in hydrogel at either pH 7.5 or 8.2. Asterisks indicate that log RCI values were significantly higher in with the wild type relative to the T6SS2 mutant (Student’s *t* test, *P < *0.0001). ns, not significant (*P > *0.05). Each experiment was performed three times, and combined data are shown (*n* = 12). Error bars indicate SEM. Download FIG S3, PDF file, 0.018 MB. Copyright © 2021 Speare et al. 2021 Speare et al. https://creativecommons.org/licenses/by/4.0/ This content is distributed under the terms of the Creative Commons Attribution 4.0 International license.

### pH-dependent phenotypes map onto T6SS2 phylogeny.

To assess the relationship between pH-dependent aggregate size and/or killing phenotypes with strain phylogeny, a consensus phylogenetic tree was built using four concatenated housekeeping genes as described previously (15). This housekeeping phylogeny did not show a clear pattern of pH-dependent or -independent hydrogel phenotypes: pH-dependent or -independent strains are found throughout the tree and do not constitute a monophyletic group. This observation does not provide strong support for the hypothesis that pH-dependent or -independent phenotypes track with housekeeping gene phylogeny in the V. fischeri strains examined (Fig. S4).

10.1128/mSphere.01288-20.7 FIG S4 pH-dependent phenotype does not map with strain phylogeny. Maximum-likelihood tree inferred with a TIM3+I+Г model of evolution for the concatenated gene fragments *recA*, *mdh*, *katA*, and *pyrC*. In this reconstruction, the root connects to a clade containing the two non-V. fischeri outgroup taxa (Vibrio salmonicida LFI1238 and Vibrio logei SA6). Statistical support is represented at nodes by the following three numbers: upper left, percentage of 500 bootstrap maximum-likelihood pseudoreplicates; upper right, percentage of 1,000 bootstrap neighbor-joining pseudoreplicates; bottom middle center, percentage of 1,000 bootstrap maximum-parsimony pseudoreplicates. Statistical support values are listed only at nodes where more than 2 methods generated support values of ≥60%. Because of a lack of space, some support values have been listed either immediately to the right of their associated nodes and/or are marked with uppercase letters (A, B, and C) in the phylogram. Circle color indicates the aggregation and/or killing phenotype in hydrogel (dark purple, pH-independent aggregation and/or killing; light purple, pH-dependent aggregation and/or killing). The black bar represents 0.1 substitution/site. Download FIG S4, PDF file, 0.088 MB. Copyright © 2021 Speare et al. 2021 Speare et al. https://creativecommons.org/licenses/by/4.0/ This content is distributed under the terms of the Creative Commons Attribution 4.0 International license.

**FIG 7:**
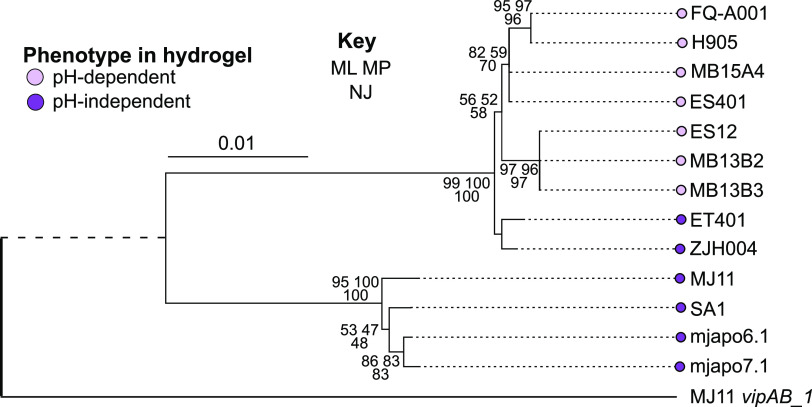
pH-dependent phenotype maps with T6SS2 phylogeny. A maximum-likelihood phylogenetic tree constructed using two concatenated T6SS structural genes (*vipA* and *vipB*) from chromosome II of 13 V. fischeri strains with an outgroup of *vipAB* genes from chromosome I of strain MJ11 (MJ11 *vipAB_1*). Circle color indicates the aggregation and/or killing phenotype of the strains in coincubations with ES114 in hydrogel (dark purple, pH-independent phenotype; light purple, pH-dependent phenotype). Statistical support is represented at nodes by the following three numbers: upper left, percentage of 1,000 bootstrap maximum-likelihood pseudoreplicates; upper right, percentage of 1,000 bootstrap maximum-parsimony pseudoreplicates; bottom, percentage of 1,000 bootstrap neighbor-joining pseudoreplicates. Nodes without support values had bootstrap values less than 50. The bar represents a branch length of 0.01 substitution per site; for clarity, outgroup branch length is not to scale.

Because the ability to make large aggregates correlates with the presence of the T6SS2 GI, we hypothesized that the evolution of genes in the T6SS2 GI might provide more insight into the evolution of these pH-dependent or -independent phenotypes. To test this hypothesis, we mapped the aggregation/killing phenotypes onto a maximum-likelihood phylogenetic tree constructed by concatenating two T6SS2 genes (*tssBC/vipAB*); *vipA* and *vipB* encode the proteins required for constructing the T6SS sheath and have been used to examine T6SS evolution (62). The *vipAB* gene tree revealed that pH-dependent and -independent phenotypes are concordant with T6SS2 structural gene evolution in the 13 V. fischeri strains analyzed (Fig. 7). Specifically, the seven pH-dependent strains cluster together and form a distinguishable subclade (Fig. 7). Furthermore, the presence of the distal pair of pH-independent strains (ET401/ZJH004) and a separate clade containing a quartet of pH-independent strains (MJ11, SA1, mjapo6.1, and mjapo7.1) is suggestive of an evolutionary hypothesis whereby the pH-dependent aggregation/killing phenotype may have arisen from an ancestral population that could aggregate and kill competitors independent of pH condition.

Because this work focused on conditions controlling T6SS2 competition, the mechanism behind enhanced aggregation observed in pH 6.5 and 7.5 in hydrogel remains unclear. Our data reveal that enhanced aggregation correlates, and may have evolved concordantly, with the T6SS2 GI, suggesting that factors facilitating enhanced cell-cell contact may be encoded within the GI. Future investigation should examine how inhibitor cells initiate contact with target cells to engage in T6SS2 competition.

### Conclusion.

**FIG 8:**
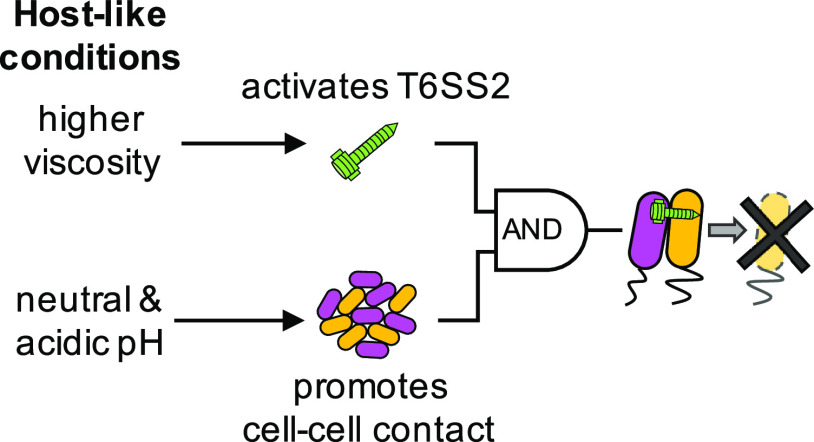
Conceptual model for how host-like conditions are required for T6SS2-mediated competition among *E. scolopes* isolates. Lethal strains (magenta) isolated from *E. scolopes* light organs require host-like conditions to outcompete a nonlethal target strain (yellow). Higher viscosity activates T6SS2 gene and protein expression, sheath assembly, and T6SS2 function while lower pH (pH 6.5 and 7.5) promotes the necessary cell-cell contact for T6SS killing. Both of these conditions must be met for a lethal *E. scolopes* isolate to outcompete a target strain.

This work underscores the role of host-specific cues in modulating competitive mechanisms and highlights the importance of including a variety of strains in functional analyses, as closely related strains isolated from similar ecological niches can show variable phenotypic responses to the same condition. Based on the above results, we propose an expanded model for T6SS2-mediated competition during colonization of the squid light organ. Juvenile *E. scolopes* squid hatch aposymbiotically and are colonized by free-living V. fischeri from the water column. As V. fischeri cells transition from planktonic to host-associated states, they experience a dramatic increase in environmental viscosity and decrease in ambient pH, from pH 8.2 in the water column to pH 6.5, as they aggregate in a mucus matrix on the light organ surface, and pH 7.5 within the crypt lumen. Our data reveal that higher-viscosity and lower-pH conditions, relative to seawater, serve as cues to activate T6SS2 function, similar to a “coincidence detector” (Fig. 8). In biological systems, coincidence detectors integrate two distinct signals that are required for a single output function (63). In this case, although the molecules that make up this coincidence detector are not yet known, host-like viscosity activates T6SS2 expression (41) and host-like pH promotes the necessary, biologically mediated cell-cell contact for T6SS2 killing. Because both of these conditions are met within the *E. scolopes* light organ, if incompatible strain types initially cocolonize the same crypt, the T6SS2+ strain can eliminate the competitor strain, resulting in a crypt that is colonized by only the T6SS2+ strain type (15).

## MATERIALS AND METHODS

See Table S2 in the supplemental material for the strains, plasmids, and oligonucleotides used in this study, and see Text S1 for growth curves and visualization of VipA-GFP sheaths.

10.1128/mSphere.01288-20.1 TEXT S1 Supplemental methods including growth curves, visualization of VipA-GFP sheaths, quantification of aggregates and single cells, and housekeeping phylogenetic analysis. Download Text S1, DOCX file, 0.02 MB. Copyright © 2021 Speare et al. 2021 Speare et al. https://creativecommons.org/licenses/by/4.0/ This content is distributed under the terms of the Creative Commons Attribution 4.0 International license.

10.1128/mSphere.01288-20.3 TABLE S2 Strains, plasmids, and oligonucleotides. Download Table S2, DOCX file, 0.02 MB. Copyright © 2021 Speare et al. 2021 Speare et al. https://creativecommons.org/licenses/by/4.0/ This content is distributed under the terms of the Creative Commons Attribution 4.0 International license.

### Media and growth conditions.

V. fischeri strains were grown in LBS medium with either 20 g/liter NaCl (790 mosM) or 35 g/liter (1,300 mosM) at 24°C (64). Medium pH was obtained by preparing stocks of 1 M buffer at each desired pH, using bis-Tris and HCl for pH 6.5 and 7.5 and Trizma base and Trizma HCl for pH 8.2 media. LBS medium at each pH was prepared using the respective 1 M buffered stock; pH of the desired medium was then confirmed using a Denver Instrument UltraBasic pH benchtop meter. Because V. fischeri can grow anaerobically using mixed-acid fermentation (65), medium pH was tested using pH test strips before and after each experiment to confirm that no change in pH occurred. We performed antibiotic selection for V. fischeri and plasmid maintenance as described previously (66–68). Hydrogel was made by supplementing LBS broth with 5% (wt/vol) polyvinylpyrrolidone (PVP).

### Coincubation assay.

Coincubation assays were performed as described previously (41, 52, 66). V. fischeri cultures were grown overnight in LBS broth supplemented with the appropriate antibiotic at 24°C. Cultures of each strain were diluted to an optical density at 600 nm (OD_600_) of 1.0, mixed in a 1:1 ratio based on OD, and 10 μl of the mixture was spotted onto agar plates or into a 12-well plate containing 1 ml of liquid or hydrogel medium and incubated at 24°C without shaking. At 0 and 12 h, strains in each coincubation were quantified by plating serial dilutions onto LBS plates supplemented with antibiotics selective for each strain.

### β-Galactosidase assay of promoter activity.

β-Galactosidase assays were performed as described previously (41, 69). Cultures of ES401 containing the reporter plasmid pAG01 were grown overnight in liquid or hydrogel medium containing kanamycin. Cultures were diluted 1,000-fold into medium containing kanamycin and grown for 12 h and cells were harvested by pelleting 0.9 ml of culture. Supernatant was discarded, and cells were frozen at −20°C for no longer than 24 h. Cell pellets were resuspended in 0.9 ml Z buffer, and β-galactosidase assays were performed using a modified Miller assay as previously described (69).

### Fluorescence microscopy and aggregate quantification.

Single-cell fluorescence microscopy was performed as described previously (15, 41). To visualize hydrogel monocultures or cocultures, V. fischeri strains containing either pVSV102 (*gfp*^+^) or pVSV208 (*dsRed*^+^) were incubated in hydrogel for 12 h, 5 μl was spotted onto a glass slide and imaged with a 60×/1.3 numerical aperture oil Ph3 lens objective (41). Images were captured with an Olympus BX51 microscope outfitted with a Hamamatsu C8484-03G01 camera using MetaMorph software. The estimated average aggregate size was determined by using the “image/adjust/threshold” and “analyze particles” commands to calculate the area of each particle. Particles larger than or equal to the area of three cells (4.5 μm^2^) were defined as aggregates, and particles less than the area of three cells were defined as single cells. The estimated average aggregate size was calculated by taking the average of the aggregate fraction for each field of view. The percent of single cells in a culture was obtained by dividing the area of the single cell fraction by the single cell and aggregate fractions as described previously (41). Additional detail on aggregate and single cell quantification can be found in Text S1.

### Preparation of antibiotic- or heat-killed cultures.

To perform experiments with dead ES401 and ES114 cells, cultures of each strain containing pVSV102 (*gfp*^+^) were initially grown in low-viscosity liquid containing kanamycin and diluted to an OD_600_ of 1.0. Heat-killed cells were prepared by incubating cells on a 50°C heat block for 1 h and then incubated at room temperature for 2 additional hours. Antibiotic-killed cells were pelleted by centrifugation at 10,000 rpm for 3 min, resuspended in LBS supplemented with 2.5 μg/ml chloramphenicol, and incubated at room temperature for 3 h. Heat- and antibiotic-killed cells were then concentrated 100-fold by centrifugation. Ten microliters of each cell type (live, heat-killed, or antibiotic-killed) was then spotted into wells containing 1 ml of liquid or hydrogel medium at pH 8.2 or 7.5, supplemented with 2.5 μg/ml chloramphenicol for antibiotic-killed treatments, and incubated at 24°C without shaking. At 12 h, cultures were imaged with a 60×/1.3 oil Ph3 objective lens; estimated average aggregate size and the percentage of single cells were calculated as described above.

### Phylogenetic analyses.

To perform the housekeeping phylogenetic analysis, multilocus phylogenetic analyses were performed using partial sequences of four loci: *recA*, *mdh*, *katA*, and *pyrC*. Published sequence data for 31 total *Vibrio* isolates were collected, combined into a single concatenated sequence (ordered *recA mdh katA pyrC*; approximately 2,880 nucleotides), and aligned with ClustalX 2.1 (more detail in the supplemental methods) (70).

To perform the T6SS2 phylogenetic analysis a multilocus T6SS2 phylogenetic analysis was performed using sequences of two loci: *vipA* and *vipB*. Published sequence data and sequence data retrieved from draft genomes of 13 total Vibrio fischeri strains were collected and combined into a single concatenated sequence (ordered *vipA vipB*; approximately 2,000 nucleotides). Concatenated *vipAB* sequences were aligned (ClustalW via MEGAX [70]) and phylogenetic reconstructions assuming a tree-like topology were created with MEGAX (71) via three methods: maximum likelihood (ML), maximum parsimony (MP), and neighbor joining (NJ). ML and MP reconstructions treated gaps as missing. Via MEGAX, the most optimal evolutionary model determined by the Bayesian information criterion (and the corrected Akaike information criterion) was the Tamura 3-parameter model with a certain fraction of invariable sites (T92+I). This model was used to construct ML and NJ reconstructions with MEGAX (tree inference was applied heuristically via the nearest-neighbor-interchange [NNI] method without a branch swap filter for 1,000 bootstrap replications) while MP was done in MEGAX with the following parameters: (tree inference was done via the subtree-pruning-regrafting [SPR] searching using random-sequence addition and 10 replicates; test of phylogeny was performed for 1,000 bootstrap replications using a nucleotide substitution model). Phylogenetic trees were visualized with MEGAX, and the final tree was edited for publication with Inkscape 1.0.

### Data availability.

Newly amplified *vipA* and *vipB* sequences have been deposited in the GenBank database (Biosample accession no. SAMN19798079 to SAMN19798082).
