# Supplementary material for: Host-Like Conditions Are Required for T6SS-Mediated Competition among Vibrio fischeri Light Organ Symbionts
Source: mSphere. 2021 Jul 21;6(4):e01288-20. doi: 10.1128/mSphere.01288-20 (PMC8386388; doi:10.1128/mSphere.01288-20)
Supplement: TABLE S1 [file msphere.01288-20-st001.docx]

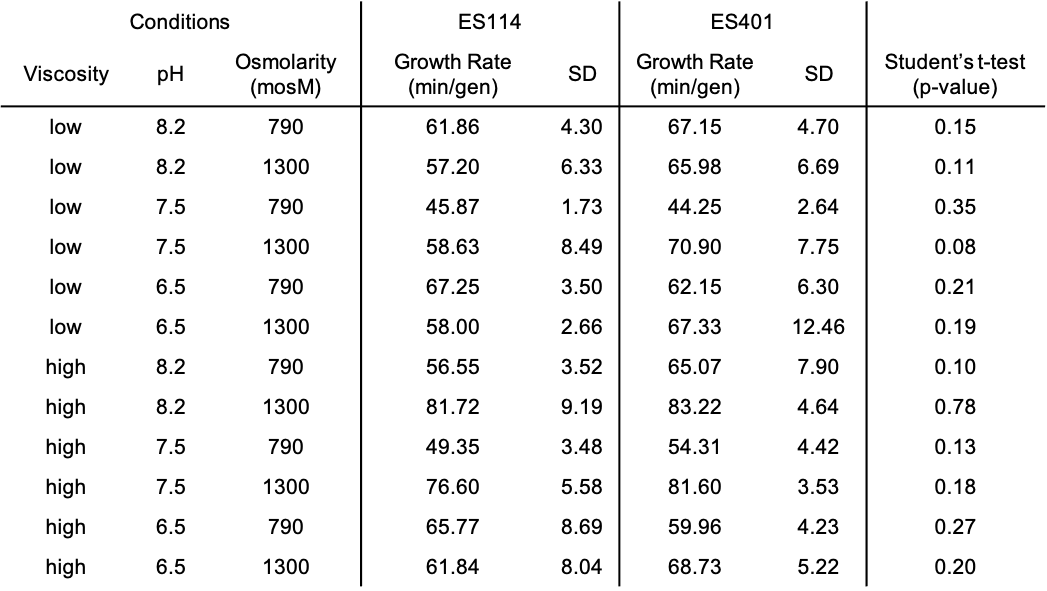
**Supplemental Table S1.** Strain growth rates in environmental and host-like conditions. Growth rates were calculated from three independent experiments with four biological replicates per experiment (n=12). There were no significant differences in ES114 vs ES401 growth rates in any given condition (Student’s t-test, column 8).
