# Supplementary figures and images for: Host-Like Conditions Are Required for T6SS-Mediated Competition among Vibrio fischeri Light Organ Symbionts
Source: mSphere. 2021 Jul 21;6(4):e01288-20. doi: 10.1128/mSphere.01288-20 (PMC8386388; doi:10.1128/mSphere.01288-20)

| pH  | Genotype          | Killing phenotype |
|-----|-------------------|-------------------|
| 7.5 | ○ Wild type       | pH-dependent      |
| 8.2 | △ <i>tssF_2</i> - | pH-independent    |

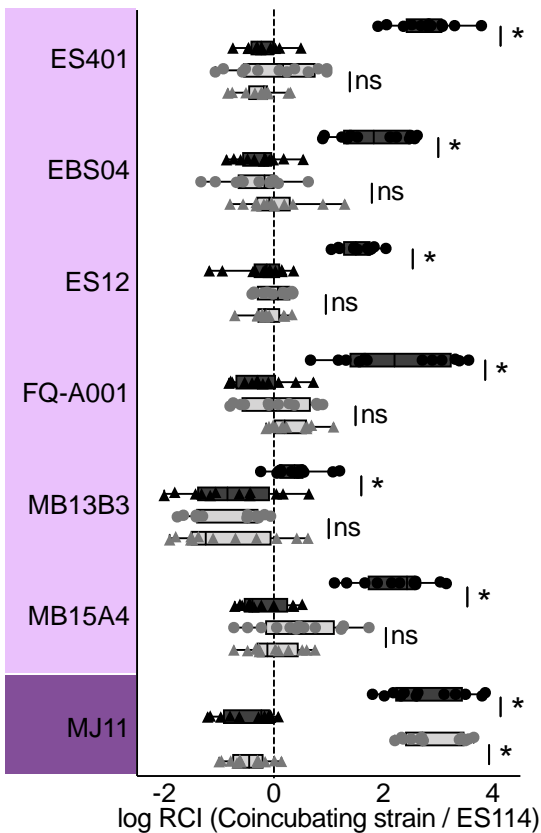

Supplement: FIG S3 [file msphere.01288-20-sf003.pdf]

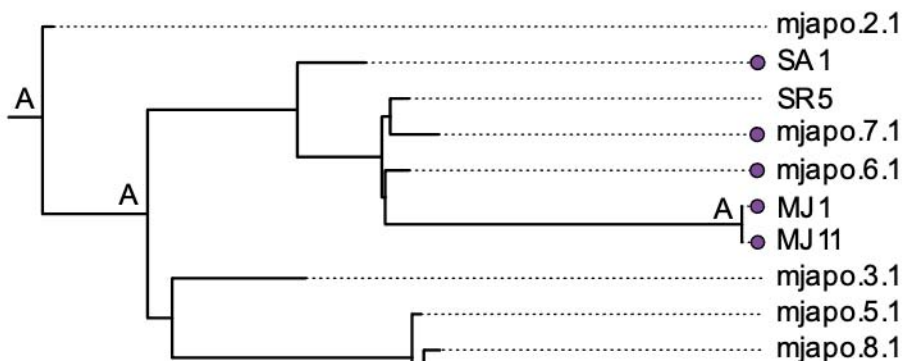

### Phenotype in hydrogel

- pH-dependent
- pH-independent

### Key

|    |    |   |   |     |     |
|----|----|---|---|-----|-----|
| ML | MP |   |   |     |     |
|    | NJ | A | = | 100 | 100 |
|    |    |   |   | 100 |     |
|    |    | B | = | 77  | 93  |
|    |    |   |   | 80  |     |
|    |    | C | = | 67  | 82  |
|    |    |   |   | 67  |     |
|    |    | D | = | 89  | 97  |
|    |    |   |   | 86  |     |

0.01

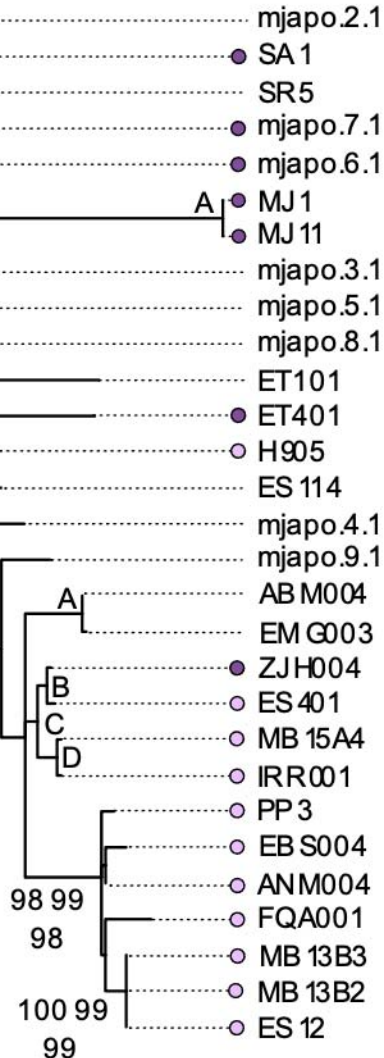

Supplement: FIG S4 [file msphere.01288-20-sf004.pdf]
