# Supplementary material for: Host-Like Conditions Are Required for T6SS-Mediated Competition among Vibrio fischeri Light Organ Symbionts
Source: mSphere. 2021 Jul 21;6(4):e01288-20. doi: 10.1128/mSphere.01288-20 (PMC8386388; doi:10.1128/mSphere.01288-20)
Supplement: TEXT S1 [file msphere.01288-20-s0001.docx]

**Supplemental Methods**

**Growth Curves.** Growth curves were performed in a 96-well Tecan plate reader and cultures were grown for 24 hours at 24C. To prepare cells for each growth condition, individual colonies were picked from LBS agar plates and grown in liquid medium with the same osmolarity and pH as the growth condition overnight. Cultures were then normalized to an OD 1.0 and 2 ul of this culture was used to inoculate each well. Growth rates of each strain in each condition were calculated by calculating the generation time for each replicate and averaging across replicates. Generation time (G) was defined as the time (t) per generation (n). First a time interval was selected in which bacteria were growing exponentially. Next, the number of generations (n) was calculated by multiplying 3.3 times the natural log of the OD at the end of the time interval (OD_end_) divided by the OD at the start of the time interval (OD_start_).

Generation time = time of exponential growth / 3.3* ln(OD_end_ / OD_start_)

G (min) = t(min) /n

n = 3.3 x ln(OD_end_ / OD_start_)

**Visualization of VipA-GFP sheaths.** Cultures of ES401 carrying the IPTG-inducible VipA_2-GFP expression vector (pSNS119) were grown in LBS liquid medium supplemented with kanamycin and 0.5 mM isopropyl-β-D-1-thiogalactopyranoside (IPTG) overnight. Cultures were then diluted 1:100 into fresh medium supplemented with kanamycin and IPTG and incubated for 2 hours. 5 ul of each culture was spotted onto a glass slide and imaged with a 100X/1.3 Oil Ph3 objective lens [1].

**Quantification of aggregates and single cells**. To perform aggregate and single cell quantification, cells were grown in LBS hydrogel for 12 hour at 24C without shaking. 5 ul of each culture was spotted onto a glass slide, covered with a cover slip, and imaged with a 60X/1.3 Oil Ph3 objective lens within 15 minutes. Images were captured with an Olympus BX51 microscope outfitted with a Hammatsu C8484-03G01 camera and were analyzed using Fiji. The background was uniformly subtracted from all images using the Subtract background command. Next, each image was thresholded using the Image🡪Adjust🡪Threshold command to divide the pixels into bins composed of either foreground particles (cells) or background (non-cells). Areas of the image that were hazy rather than being defined cells were attributed to cells that were out of focus and were excluded in this thresholding step; this was done uniformly across all images.The size of each particle was then calculated using the Tools🡪Analyze Particles command, with a pixel size (pixel^2) of 0.01-infinity. This results in a table with the area of each particle. Particles were then sorted by area based on the area of three *V. fischeri* cells (~4.5 μm^2^); this area value was calculated and adjusted for each strain. Particles that were smaller than the area of three *V. fischeri* cells were considered single cells, while particles equal to or larger than the area of three cells were considered aggregates. This quantification does not take into account the 3-D nature of aggregates in hydrogel and only calculates the area therefore, these measurements are likely an underestimation of aggregate size.

**Housekeeping Phylogenetic Analysis.** A multi-locus phylogenetic analysis was performed using partial sequences of four loci: *recA*, *mdh*, *katA*, and *pyrC*. Published sequence data of 31 total *Vibrio* isolates were collected, combined into a single concatenated sequence (ordered *recA mdh katA pyrC* – approximately 2880 nucleotides), and aligned with ClustalX 2.1 [2]. Phylogenetic reconstructions assuming a tree-like topology were created with three methods: maximum parsimony (MP); maximum likelihood (ML); and neighbor joining (NJ) in a manner similar to previously described methods [3, 4]. MP reconstructions were performed by treating gaps as missing, searching heuristically using random addition, tree-bisection reconnection with a maximum of 8 for swaps, and swapping on best only with 1000 repetitions. (For ML and NJ analysis, likelihood scores of 1500+ potential evolutionary models were evaluated using the corrected Akaike Information Criterion, the Bayesian Information Criterion, and Decision Theory (Performance Based Selection) as implemented by jModelTest2.1. [5]. For Bayesian and Decision Theory evaluations, the most optimal evolutionary model was a transition model including six free parameters with a gamma distribution and proportion of invariant sites used to model rate heterogeneity over alignment sites. (TIM3+I+Г).

ML reconstruction was implemented via PAUP*4.0a163 [6] (*and Other Methods)
by treating gaps as missing, searching heuristically using random addition, tree-bisection reconnection for swaps, and swapping on best only with 1000 repetitions. Neighbor-joining methodology was completed with MEGAX 10.1.8 [7] using the Maximum Composite Likelihood method [8] with rate variation among sites modeled with a gamma distribution (shape parameter = 1). For ML, MP, and NJ analyses, the statistical confidence in the topology of each reconstruction was assessed using 500, 1000, and 1000 bootstrap replicates, respectively. Phylogenetic trees were visualized with FigTree 1.4.3 (http://tree.bio.ed.ac.uk/software/figtree); the final tree was edited for publication with Inkscape 1.0 (4035a4f, 2020-05-01) (http://inkscape.org/) and GIMP 2.10.8 (<http://www.gimp.org/>).

**References**

1. Speare, L., et al., *Bacterial symbionts use a type VI secretion system to eliminate competitors in their natural host.* Proc Natl Acad Sci U S A, 2018. **115**(36): p. E8528-E8537.

2. Larkin, M.A., et al., *Clustal W and Clustal X version 2.0.* bioinformatics, 2007. **23**(21): p. 2947-2948.

3. Mandel, M.J., et al., *A single regulatory gene is sufficient to alter bacterial host range.* Nature, 2009. **458**.

4. Wollenberg, M.S. and E.G. Ruby, *Phylogeny and fitness of Vibrio fischeri from the light organs of Euprymna scolopes in two Oahu, Hawaii populations.* The ISME journal, 2012. **6**(2): p. 352-362.

5. Darriba, D., et al., *jModelTest 2: more models, new heuristics and parallel computing.* Nature methods, 2012. **9**(8): p. 772-772.

6. Swofford, D.L. and J. Sullivan, *Phylogeny inference based on parsimony and other methods using PAUP*.* The Phylogenetic Handbook: A Practical Approach to DNA and Protein Phylogeny, cáp, 2003. **7**: p. 160-206.

7. Stecher, G., K. Tamura, and S. Kumar, *Molecular evolutionary genetics analysis (MEGA) for macOS.* Molecular Biology and Evolution, 2020. **37**(4): p. 1237-1239.

8. Tamura, K., M. Nei, and S. Kumar, *Prospects for inferring very large phylogenies by using the neighbor-joining method.* Proceedings of the National Academy of Sciences, 2004. **101**(30): p. 11030-11035.
