# Supplementary material for: Host-Like Conditions Are Required for T6SS-Mediated Competition among Vibrio fischeri Light Organ Symbionts
Source: mSphere. 2021 Jul 21;6(4):e01288-20. doi: 10.1128/mSphere.01288-20 (PMC8386388; doi:10.1128/mSphere.01288-20)
Supplement: TABLE S2 [file msphere.01288-20-st002.docx]

**Supplemental Table S2**. Strains, Plasmids, Oligo table

| **Strain** | **Host** | **Reference** |
| --- | --- | --- |
| ABM004 | *Euprymna scolopes* | Speare *et al.,* 2018 |
| ANM001 | “” | “” |
| EBS004 | “” | “” |
| EMG003 | “” | “” |
| ES114 | “” | Boettcher and Ruby 1990 |
| ES12 | “” | “” |
| ES401 | “” | Lee 1994 |
| ET101 | *E. tasmanica* | Nishiguchi 2002 |
| ET401 | *“”* | “” |
| H905 | *E. scolopes* | Lee and Ruby 1992 |
| IRR01 | *“”* | Speare *et al.,* 2018 |
| MB13B2 | *E. scolopes* | Wollenberg and Ruby 2009 |
| MB13B3 | *“”* | “” |
| MB15A4 | *“”* | “” |
| MJ11 | *Moncentris japonica* | Ruby and Nealson 1976 |
| MJ1S | *“”* | Bose and Stabb 2011 |
| mjapo2.1 | *“”* | P.V. Dunlap and Mandel *et al.,* 2009 |
| mjapo3.1 | *“”* | “” |
| mjapo4.1 | *“”* | “” |
| mjapo5.1 | *“”* | “” |
| mjapo6.1 | *“”* | “” |
| mjapo7.1 | *“”* | Dunlap *et al.,* 2007 |
| mjapo8.1 | *“”* | P.V. Dunlap and Mandel *et al.,* 2009 |
| mjapo9.1 | *“”* | “” |
| PP3 | *Planktonic / E. scolopes* | Lee and Ruby 1992 |
| SA1 | *Sepiola affinis* | Fidopiastis *et al.,* 1998 |
| SR5 | *S. robusta* | “” |
| ZJH004 | *E. scolopes* | Speare *et al.,* 2018 |
| **Strain** | **Relevant Characteristics** | **Reference** |
| ANS2098 | FQ-A001 with *tssF_2* disruption (Erm^R^) | Speare *et al.,* 2018 |
| ANS2100 | ES401 with *tssF_2* disruption (Erm^R^) | Speare *et al.,* 2020 |
| LAS003 | EBS004 with *tssF_2* disruption (Erm^R^) | Speare *et al.,* 2018 |
| LAS004 | MJ11 with *tssF_2* disruption (Erm^R^) | This Study |
| LAS007 | MB13B3 with *tssF_2* disruption (Erm^R^) | Speare *et al.,* 2018 |
| LAS008 | MB15A4 with *tssF_2* disruption (Erm^R^) | “” |
| LAS009 | ES12 with *tssF*_*2* disruption (Erm^R^) | This Study |
| **Plasmids** |  |  |
| pAG01 | *P_hcp_2_-lacZ* promoter reporter; *oriV_R6k_*_γ_, *oriV_pES12_, oriT, Kn^R^* | Speare *et al.,* 2020 |
| pAS2038 | *tssF_2* disruption vector; *oriV_R6k_*_γ_, *oriT, Erm^R^* | Speare *et al.,* 2018 |
| pEVS104 | conjugative helper, *oriV_R6k_*_γ_, *oriT, Kn^R^* | Stabb and Ruby 2002 |
| pSNS119 | *vipA_2-gfp* fusion vector; *oriV_R6k_*_γ_, *oriV_pES213_, oriT, Kn^R^* | Speare *et al.,* 2018 |
| pVSV102 | *gfp+, oriV_R6k_*_γ_, *oriV_pES213_, oriT, Kn^R^* | Dunn *et al.,* 2006 |
| pVSV208 | *dsRed+,* *oriV_R6k_*_γ_, *oriV_pES213_, oriT, Cm^R^* | “” |
| **Oligonucleotides^a^** |  |  |
| AS1146 | TAGGTACCCTGATGTTGAACGCTTATTAG | Speare *et al.,* 2018 |
| AS1147 | ATGCATGCAGATACTTGATTGTTATGCG | “” |
| *recA* *outer-F* | GACGATAACAAGAAAAAAGCACTGG | Wollenberg *et al.,* 2012 |
| *recA* outer-R | CGTTTTCTTCAATTTCWGGAGC | “” |

^a^ Restriction sites are underlined
